# Health care staff support for mothers in NICU: a focused ethnography study

**DOI:** 10.1186/s12884-021-03991-3

**Published:** 2021-07-21

**Authors:** Reza Negarandeh, Hadi Hassankhani, Mahnaz Jabraeili, Mohammad Abbaszadeh, Amy Best

**Affiliations:** 1 Nursing and Midwifery Care Research Center, Tehran University of Medical Sciences, Tehran, Iran; 2 Center of Qualitative Studies, Department of Medical Surgical Nursing, School of Nursing and Midwifery, Tabriz University of Medical Sciences, Tabriz, Iran; 3 School of Nursing and Midwifery, Tabriz University of Medical Sciences, Tabriz, Iran; 4 Department of Sociology, University of University, Tabriz, Iran; 5 Campus Teacher, School of Nursing, Massey University Wellington, Wellington, Australia

**Keywords:** Mother, Support, Nurse, Ethnography, Neonatal Intensive Care Unit

## Abstract

**Background:**

Mothers of premature newborns in the neonatal intensive care unit (NICU) have complex needs and require a significant amount of support during the NICU admission. However, little is known about mothers' support needs in the NICU. This study aimed to explore health care staff and mothers' experiences of meeting the mothers support needs in the NICU.

This study aimed to explore health care staff and mothers' experiences of meeting the mothers' support needs in the NICU**.**

**Methods:**

A focused ethnographic approach was adopted. Observations and interviews with 21 mothers, 18 nurses, and five physicians were undertaken over a seven months period. Qualitative data analysis was conducted using the Roper and Shapira (2000) five-step framework.

**Result:**

Two main themes of “insufficient provision of the mothers' support needs” (subthemes: inadequate accompany of the mothers in care, assigning monitoring and care to the mothers, inadequate sharing of medical the information) and “supporting the mothers in certain circumstances” (subthemes: reassuring the mothers, supporting the mothers with reduced functional capacity, providing information) were obtained.

**Conclusion:**

The mothers experienced a gap between expected and actual support provided by health care staff. Although, the health care staff believed that mothers' support was a necessity, it was not their main concerns, and they considered workload as a barrier for the mothers support in the NICU.

## Background

The Neonatal Intensive Care Unit (NICU) provides intensive and specialized therapeutic care for complicated newborns [1]. Most premature newborns, and full-term newborns with particular need of care such as congenital anomaly, maternal diabetes, hypertension, and necrotizing enterocolitis require hospitalization in the NICU [2].

According to previous studies, mothers experience a moderate level of stress and anxiety during the hospitalization of their newborn [3–5]. The impact of preterm birth and subsequent hospitalization in the NICU on maternal mental health is significant. Mothers are at increased risk of postpartum depression, anxiety, and post-traumatic stress disorder [2, 5].

Four major NICU environment stressors were identified, including stressors of physical environment, newborn appearance and behavior, parent- newborn relationship, and staff [6]. Alterations of parental role and newborn appearance and behavior are the most important sources of stress in the NICU [7, 8]. Furthermore, the inability to perform parental roles could be result in their anxiety and acute grief [9].

Various maternal and infant characteristics have an impact on maternal stress. There are significant positive relationships between maternal stresses with primiparous pregnancies, marital status, distance from home, newborn gestational age, newborn’s gender, length of stay, and newborn medical condition [4, 8]. Trained health care staff could help the mothers to participate in their newborn care and reduce the mothers’ stress by giving information [10]. Ability to talk daily with the health worker team, feeling that the hospital staff is taking good care of the patient, knowing what will happen to the patient, providing answers accurately and indeed questions, were among the most essential needs of mothers in NICU[11]. A systematic review in Iran showed that the need for assurance, awareness, closeness, support, and comfort was of the most essential needs of parents [12]. Generally, the need for support is one of the main findings of studies derived from the experiences of parents of premature newborns admitted to the neonatal intensive care unit [11, 13–15]. In order to minimize mother–child relationship failure following the newborn’s immediate hospitalization, health care staff support for mothers is necessary in the NICU [14].

The social support includes family, friends, Spirituality, and health care professionals [16]**.** Health care professionals can establish caring relationships. Sharing information, paying attention to supporting psychologically and physically, involving mothers in their infant’s care reduce maternal stress [17]**.** Supporting the parents results in decreasing their anxiety, increasing their awareness, growth of “self-confidence to their capabilities” in taking care of the newborn [18], and finally, reduction of hospitalization period of the newborn and increasing parents' satisfaction [19]. Once discharged from the hospital, health care professionals’ support is less available for mothers; instead, informal support plays an essential role within the family [20].

Therefore, it is essential that health care workers must provide adequate support to help parents cope with the stress of newborn hospitalization and improve their ability and confidence in the care of newborns [21]. Given that nurses are care providers, they have a critical role in providing the support [22].

The cultural norms of care environments can affect maternal experiences and their support needing staff roles in the NICUs across different countries [23]. Culture is defined as the set of rules of life they have learned. These rules tell group members how to behave, and how to respond to the world around them. Culture has two main dimensions of cognition and behavior. The cognitive dimension includes the values, beliefs, ideas, and knowledge of members of a group of people living together, and the behavioral dimension includes what they eat, wear, speak, and act on [24].

Most of the studies have focused on the mothers’ experiences in NICU [7, 25, 26], and there is not enough information about the experiences of health care staff alongside mothers on support for the mothers in the NICU.

This study aimed to explore health care staff and mothers' experiences of meeting the mothers' support needs in the NICU**.**

## Methods

This paper is part of a study titled “The Neonatal Ward Culture in Collaborative Maternal Care”, which was conducted through focused ethnography. In this study, a social phenomenon affecting maternal involvement such as mutual trust, support, communication between health care workers and mothers were identified.

### Settings and sampling

The study setting was a NICU (level II) in Tabriz, Iran, Which is the primary pediatric’ referral center for full-term and premature newborns located in the East Azarbaijan Province, in the North West of Iran.

The physical space of the level two NICU consisted of a large hall equipped with 24 incubators. There was an average of three nurses on each shift, which provided nurse-to-patient ratio of 1:5–8. The nurse-to-patient ratio is one-to-one for newborns requiring intensive care [27].

Twenty-one mothers of newborns (13 mothers of term newborns and eight mothers’ of late preterm newborns) and 18 nurses (16 nurses’ circular shifts and two nurses stable shift) and five physicians were selected using a purposeful sampling method. All staff was verbally invited to participate in the study.

The mothers who had continues presence beside their newborns hospitalized in the NICU for more than one week, and nurses and physicians who had at least six months’ work experiences in the NICU and involved in the care of the newborns were enrolled in the study.

The study was approved by the Research Ethics Committee Tabriz University of Medical Sciences (TBZMED.REC > 2016.789). At the beginning of the study, the principal investigator (PI) explained the study to participants (mothers & staff) who signed an informed consent approved by the research ethics committee.

The mothers’ presence requirement of this NICU was the ability to provide primary care for their newborn. Mothers were responsible for breastfeeding, sucking practice, hygiene cares, skin care, and comfort cares such as massage and settling the newborn. Mothers were also involved in specialized neonatal care such as oral drug administration and gavage feeding depended on the mother’s confidence and capability.

### Data collection

Data collected in January-July 2017 through ethnographic fieldwork methods of participant observation and ethnographic interviews. The principal investigator spent different times, on different days and different shifts for observing events and interactions between participants for 7 months. Due to the long-term presence of the researcher in the NICU, all staff acted and interacted regularly.

The descriptive, focused, and selective observation was performed. The objective of descriptive observations was the awareness of the variety of events that occur during a normal day. For example, PI sat in the chairs in the unit and tried to describe everything she saw: the patients’ admission, the procedure of evaluation, the comments made by mothers, and actions of the NICU staff. In focused observation, collaborative observation approach [24] was used at the newborn's bedside which the researcher observes while also participating in care. Mothers' observation was while the mother caring for newborns and staff observation was during their interactions with mothers close enough to ask and share information to clarify why the work was done.

Verbal and non-verbal cues and behaviors, were considered during observation. The selective observation was typically focused on a specific feature of the Participants' activity (touching, time spent with the patient, etc.), or behavior (smiling, talking, etc.) actions.

The observation ended when the researcher did not found new data, the research question was answered, and data saturation had been achieved.

Interviews (formal and informal) were conducted by the first author. Informal interviews with mothers and health staff were conducted after the observations which was focused on clarifying ambiguities observed in the fieldwork and was transcribed promptly. For example, when PI observed a nurse who refused to help a mother in diaper changing, asked her to explain the reasons of not helping the mother.

Formal interviews with the mothers focused on their experience of having a newborn in the NICU and their support needs. Formal interviews with the staff focused on their experience of caring for newborns, providing support to mothers, as well as their personal and professional beliefs, attitudes, perspectives, and values concerning the care of mothers. Interviews were recorded and transcribed verbatim. Interviews were stopped if a participant did not wish to continue. Formal face-to-face interview was conducted by PI with health staff and mothers lasted between 30–60 min. Voice recorder used for recording formal interviews with the consent of the participants. Interviews with staff were done in a quiet room near the unit, and mothers were interviewed bedside their newborn for the comfort of them.

The interview began with an open-ended question. Nurses were first asked to "describe their experiences of supporting mothers in neonatal care." This was then followed up with questions such as "what do you think about supporting mothers?" "What kind of caring support do you think mothers need?" “Are there opportunities and constraints for care to support mothers with a newborn?" “How do you meet the needs of supporting mothers with newborns?” Mothers, on the other hand, were first asked to "share their experiences of support from nurses in their neonatal care." This opening question was followed by others such as "what problems do you face while participating in newborn care?”, “Who do you see when you have a care problem?" "What help do you receive from nurses about neonatal care?" "How do staff provide you with the information you need?" “What do you expect nurses to do to support you?”.

### Data analysis

All interviews and data analysis for this study was conducted in Persian. Following data analysis, this bilingual (Persian and English), bicultural, team of nursing researchers translated the codes and emerging themes from Persian to English. When data analysis was complete, all exemplar quotes were back-translated [28].

Data analysis began at the same time as data collected. Analysis of the data was conducted using Roper and Shapira’s [24] 5-step Framework. Observations, informal conversations, are converted into field notes, and taped interviews are transcribed. Interviews and field notes were reviewed and re-read several times to gain an overall understanding, and then the analysis were proceeded line by line and was assigned a meaningful code in the coding step. The second step was sorting for patterns. Similar codes were put into a group or category called basic concepts. These concepts were then grouped in similarity to form sub-themes. These sub-themes were also incorporated into the main themes. The third step was identification of outliers where negative cases that did not match the study findings were the identified and analyzed in the data. The fourth step was generalizing. Constructs and theories of the findings were compared with existing studies and theories. The fifth step was memoing. Memos were reflections that occur during all stages of data collection and analysis and provide the basis for deep and meaningful understandings of data. For example, the nurse said in the interview that mothers do not need help to change diapers and often do. **Memo**: “Do mothers also have this opinion? In the interview, mothers should be asked about this issue”. Analysis of the data was conducted by the primary author and verified by the research team. The research team included two faculty experts in qualitative research, and a faculty sociologist expert in ethnography.

### Rigor and trustworthiness

The study rigor was obtained using Lincoln and Guba’s [29] criteria. To increase the validity of the data, observation integration, interviewing for data collection, and reflexivity was used throughout the research. The researcher sought to increase the validity of the findings by prolonged and profound involvement with the data and long-term presence in the NICU.

Reliability and validity were determined by triangulation (observation & interview), reflexivity, Peer Checking, external check, and member checking.

The principal investigator for ten years had worked as a clinical instructor in the NICU, and she used reflexivity to reduce the effects of his viewpoints on the results. The PI, to reduce the impact of individual values, recorded her thoughts in diary notes before and during data collection and analysis. Peer Check was conducted by the research team, and external check was conducted by referees of the thesis. For member check, the text of the interview, primary codes, and main themes were provided to the two nurses, one physician, and the two mothers, and they were asked to compare analysis results with their own experiences, and provided their opinions.

## Results

Eighteen nurses, five physicians and twenty-one mothers participated in the study. The demographic characteristics of participants are presented in Tables 1 and 2. Two hundred and fifty hours of participant observation were undertaken over seven months. Interviews were of 30–60-min duration. Findings from this study showed that the culture of support in the NICU was one of compassionate support. Two themes emerged from the data: Insufficient provision of mothers' support needs and provision of support in special circumstances (Table 3).

**Table 1:** Participants (physicians and nurses) characteristics

**Staff**	**Sex** **N (%)**		**Age/year** **Range**	**Marital Status** **N (%)**		**Education**	**Work Experience** **(y) Range**	**Job Status** **N (%)**		**Shift** **N (%)**	
	**Male**	**Female**		**Married**	**Single**			**Official**	**Contractual**	**Fix**	**Rotation**
**Physician (N = 5)**	**4(80%)**	**1(20%)**	**35–45**	**5(100%)**	**0(0%)**	**Neonatal Specialist**	**10–25**	**4(80)**	**1(20)**	**5(100)**	**0(0%)**
**Nurse (N = 18)**	**0**	**8(100%)**	**24–49**	**12(67%)**	**6(33%)**	**Bachelor of Science**	**1–19**	**12(67%)**	**6(33%)**	**2(11%)**	**16(89%)**

**Table 2:** Participants (mothers and newborns) characteristics

	Characteristic	Range		N (%)
**Mother (N = 21)**	**Age (years)**	20–35		
	**Marital status**	Married		21 (100%)
	**Education level**	Primary education		4 (19.5%)
		Secondary education		11 (52%)
		Bachelor of Science		6 (28.5%)
	**Parity**	Nulliparous		16(76%)
		Multipara		5(24%)
	**Job status**	Housewife		18 (86%)
		Employed		3 (14%)
**Neonate(N = 21)**	**Birth weight (grams)**	1300–4500		
	**Gestational age (weeks)**	32–42		
	**Age on admission (days)**	1–28		
	**Length of stay (days)**	7–30		
	**Sex**	Male	10(43%)	
		Female	12(57%)	
	**Maturity**	Term	14(66.5%)	
		Preterm	7(33.5%)	
	**Diagnosis**	Malformation	7(33%)	
		Infection	6(28.5%)	
		RDS	5(24%)	
		Seizure	2(9.5%)	
		Metabolic Diseases	1(5%)

**Table 3:** Primary and subthemes of study

Primary Themes:	
**Failure to Meet the Support Needs of Mothers**	**Support in Special Circumstances**
**Subthemes:**	
Failure to Accompany the Mother in Care	Reassuring the Mother
Assigning Monitoring and Care to the Mother	Supporting Mothers with Reduced Functional Capacity
Inadequate Sharing of Medical Information	Providing Information

### Insufficient provision of mothers' support needs

Data from observations and interviews showed two things. Firstly, that mothers of neonates in the NICU had complex support needs. Secondly, that these support needs were not adequately met. Within this theme of insufficient provision of mothers' support needs, three- sub themes emerged; 1. Inadequate Accompany of the Mother in Care, 2. Assigning responsibility of care to the mother, and 3. Inadequate sharing of information.

### Inadequate accompany of the mother in care

Observations and interviews showed that all mothers needed the support of nurses due to unfamiliarity with both neonatal care and the clinical environment. The need for support was more felt for first-time mothers due to be inexperience. Support needs were also more for the mothers who lived regionally due to traveling long distances coupled with the inability to rest at home. During the first days of hospitalization, mothers who lacked the skills to care for their newborns demonstrated constant concern and worried about the quality of care they provided. One of the mothers described her first experience of gavage.

*"The first time I tried to gavage feeding, I was terrified. I was saying that maybe the milk enters the lungs and the baby suffocates."(Mother7)*

They constantly sought guidance and reassurance from nurses. However, this was complicated because nurses were often unable to provide the necessary emotional support due to time constraints and high patient loads.

One mother demonstrates her need for close guidance and support when she says:

*“I used to say just come and stand with me while I change the nebulizer water or the diaper.” (Mother 1)*

Another mother demonstrates her need for lactation support as well as reassurance when she says:

*“Because I did not know how to breastfeed and was scared to hurt my newborn, I asked the nurse, am I caring for my newborn properly?.” (Mother 3)*

Although nurses believed that supporting mothers was necessary, resource constraints, specifically staffing, meant that they could not to provide such supportive care. One nurse demonstrates the inner conflict this causes when she says:

*"Mothers, especially those who are from the country, are under significant pressure. It is difficult for us to support the mother as well as care for the newborn because we are so busy." (Nurse 14)*

### Assigning monitoring and care to the mother

It was common practice for nurses to assign the responsibility of monitoring and care to the mother. If a mother was constantly present, then the nurse was less likely to examine the newborn. Also, newborns whose mothers had previous experience of childcare appeared to receive less attention from nurses. This is because nurses had confidence inexperienced mother’s ability to perform simple care. Often in these cases, nursing presence was minimal, for example, to provide the mother with specific clinical instructions. The experienced mothers were frustrated and dissatisfied that they been assigned the primary responsibility of the care of their newborn. They were displeased that because they were experienced mothers, nurses failed to see the need to assess the newborn. This is demonstrated by one mother who said:

*“I breastfed my son in the morning, and then he went to sleep. Now he will not wake up, which is worrying. The nurse told me to knock on his feet to get up, but he fell asleep again. I feel strongly that the nurse must examine my newborn. Maybe something has happened to him.” (Mother 9)*

Nurses’ reliance on the mother’s constant presence and availability also had negatively impacted for the mother’s health. Rest for the exhausted mother was not protected. For example, if a mother was resting and her newborn began crying, the mother was expected to tend to the newborn immediately. On the other hand, lack of confidence in nursing care forced mothers to spend their rest hours in the chair beside the newborn– proximity was crucial. The following is one of the observations in this regard.

*“The mother was asleep on the chair. When I reached the top of her head, she woke up. I asked why she did not go to the mothers' room to rest. The mother said: Yesterday I came back from mothers’ room, and saw that my baby was crying too much and his face is bruised, but the nurses did not notice her crying”.*

These fears around inadequate and missed care and frustration of unprotected rest are demonstrated by the following mother.

*“When I am not present, no one cares for my newborn. When I am asleep, and my newborn is crying and waking me up early.” (Mother 16)*

### Inadequate Sharing of Medical Information

The mothers felt that medical staff, special physicians, did not spend enough time sharing clinical information. They felt that there was not sufficient time dedicated for answering their questions and concerns about the newborn. One of the mothers said:

*“The doctor visits soon and goes to another patient, and I cannot ask my questions.” (Mother15)*

Most of the mothers would seek out the physician when they had finished rounds and leave the NICU to have their questions answered. In these instances, the physician's brief answer to posed questions was not satisfactory. For example,

*“I show the physician the chest X-ray images and ask for further explanation on my newborn’s medical condition. The physician responds by simply saying his lungs are infectious. I would expect him to explain more. “(Mother5)*

The nurses also believed that the physicians’ explanation was not enough and was not understood by the mothers.

*“Doctors do not explain deeply, and their explanations at the mother's level of understanding, or use terms that they do not understand what they mean.” (Nurse7)*

Barriers to medical staff not being able to spend time communicating with family appeared to be multi-factorial including medical staff shortage, busy workloads and providing clinical treatment alongside students’ training. Physicians appeared to spend most of their time training students due to clinical demands. Sometimes prolonged training and time constraints meant they had to hurry to examine patients. If a mother had more questions, she would have to wait for the physician to finish the medical rounds. One of the physicians said:

“*Because after visiting the babies, I have to attend medical student education classes, so I have little opportunity to visit and I do not have enough time to answer all the questions of mothers.” (physician2)*

Another physician said:

“*Most tests are specialized, and the mother is not able to understand them, so I do not see the need to explain them in more detail, and as soon as the mother knows there is nothing to worry about.*” *(physician1)*

When mothers were unsatisfied with physicians sharing information and clinical explanations, they would seek further clarification from nurses. Many of these questions were clinical, related to tests or para-clinical procedures. Nurses, however, we're unable to answer many of these types of questions due to a lack of knowledge. They felt uncomfortable and stated that answering such specialized questions was outside their scope. Nurses asserted that it was a medical responsibility to answer medical questions. One nurse demonstrates this when she says:

*“The mother wants me to interpret the results of the lumbar puncture test. Well, this test is a specialized test. So, I tell the mother that she needs to ask the physician about this specialized test. We do not have the knowledge to interpret findings.”* (*Nurse 14)*

Another nurse supports this when she says:

*“We are told what the newborn’s medical problem is. The nurse is not allowed to announce the result of ultrasound. We cannot precisely interpret. It is the physician's duty to interpret the test.”* (*Nurse 3)*

### Support under special circumstances

As previously described, nurse-patient ratios appeared to be a significant contributing factor to the mothers' support needs not being met. High workloads meant they were unable to attend to the mother. However, there were some nurses did who did provide support for mothers whenever the slightest opportunity presented. These nurses exhibited empathy, kindness, and compassion towards mothers. Within this theme of ‘support under special circumstances,’ three sub-themes emerged; 1. Reassuring the mother. 2. Accompanying the mother with reduced functional capacity, and 3. Providing information.

### Reassuring the mother

The mothers of newborns who had physical abnormalities (such as hydrocephaly, cleft palate, meningomyelocele) expressed frustration and upset at facing new conditions in care. For example, the method of tying a newborn diaper that had undergone bladder exstrophy surgery is different from a healthy newborn, so a multipara mother had trouble tying her infant's diaper. In these situations, some nurses offered encouragement and assured the mother that they would support her with care. A mother whose baby had bladder exstrophy said:

*“I did not know how to tie my baby's diaper. I was crying and worried. The nurses told you do not worry, we will teach you how to do it.” (Mother12)*

The following describes an observed moment when a nurse attempts to reassure the mother of a newborn with meningomyelocele.

The mother was crying. Through her tears, she asked the nurse how to breastfeed her newborn and what to do with her back. The nurse kindly faced the mother and nodded. She responded by saying:

*"Do not worry, I will teach you how to breastfeed. I will tell you what to do. Do not worry and trust in God”.*

This nurse identified that mother's need in that particular moment and subsequently provided her with the help and reassurance she required. Some nurses would advise the mother to rest when she felt tired, assuring her that she would take care of the newborn in her absence whenever they were free.

*"I tell the mother that ‘I am here for half an hour or an hour. If your newborn wakes up, I will give him milk and wake you up if I need you.’ This enables the exhausted mother to rest for half an hour.”* (*Nurse 9)*

### Supporting mothers with reduced functional capacity

Some mothers were unable to carry out physical care due to reduced functional capacity. For example, physical function was decreased in mothers who had undergone cesarean section or had vaginal stitches. In these instances, nurses were required to provide primary care for the newborn.

*"Changing a diaper is the duty of the mother, but sometimes an ill mother, such as one post cesarean section, may not be able to attend to all of her newborn's cares. In these cases, we have to assist the mother with caring for her newborn." (Nurse 13)*

Post-natal mental health issues such as low mood, depression, anxiety, and stress also affected a mother’s ability to care for her newborn. If depressive symptoms such as disappointment, insomnia, feel guilty, etc., were evident or if a mother was stressed and, or overwhelmed about the clinical condition of her newborn, more significant from nurses was required. For example, the mother of the baby who had seizure, said to the nurse who has taught her massage:

*"I do not think I can massage my baby, because I feel it is ineffective" (observation).*

At this time, the nurses decided to take care of the newborn instead of the mother. One of the nurses said:

*“I told his mother to give the newborn massage, but she said I am hopeless, he was not getting better. I decided to give massage to the newborn.”(Nurse8)*

Another example of situations when nurses would provide more significant support to mothers was when a mother appeared to have exhausted all options of consoling her upset newborn. Often, in these situations, the mother was visibly upset. One nurse describes this when she says:

*"Most mothers have trouble calming their newborns. They get distressed that their newborn will not stop crying. We check to see if the newborns upset is due to a dirty diaper or bloating." (Nurse 14)*

### Providing information

Overall, the mothers reported that they were provided with little clinical information regarding treatment and illness. However, there was some satisfaction around communication regarding certain care practices. For example, the mother of a newborn with a cleft palate asked the physician:

*"Can I give my milk to my newborn?” The physician responded by saying, “Yes, you can breastfeed, and you have to bend over, and your nipples have to fill your newborn’s mouth. However, if you are unable to do that, then alternatively use this bottle." (Physician 4)*

Sometimes the physicians prescribed specialized care to teach mothers by nurses.

*"The physician told the resident" write teaching chest physiotherapy, strengthening sucking, massaging and feeding gavage to mother by nurses. Because we may have to discharge newborn with NGT."* (*Physician 3)*

Additionally, the nurses provided educational guidance when a mother was faced with difficulties in caring for her newborn.

*"Our mothers are afraid to give syrup to their babies, so we remind mothers to dilute medications such as multivitamins, keep their heads up, and give them slowly.”* (*Nurse 1)*

Interviews with the mothers illustrated that the mothers in these instances reported satisfaction with the nurse’s education in taking care of her newborn. For example, one mother said:

*“The nurse taught me kangaroo care and massage on the mannequin in the training class.” (Mother 9). I would pour the milk through the tube, and the nurses would say add the distilled water. They brought distilled water themselves. "(Mother 10).*

## Discussion

Based on the findings of the present study, the themes of “failure to meet the support needs of mothers” and “supporting the mothers in certain circumstances” were emerged, which will be discussed below.

Our findings suggest that in an Iranian NICU, mothers' support needs are not adequately identified, and could not be met. This failure to meet the support needs of the mothers is attributed to a multitude of factors, namely lack of human resources resulting in high patient-to-nurse ratio and a culture of not providing enough support for the mothers to participate in their newborn primary care, while nullipara mothers particularly need the support.

The findings of this study showed that, mothers in the NICU have always wanted nurses to monitor their care due to lack of experience with a newborn and their care requirements, and unfamiliarity with the clinical environment. This is supported by Bruce and colleagues. [30] They asserts that mothers feel significant pressure taking care of their newborns, and need ongoing communication with the nurses for participatory care. Mothers' need support and guidance from nurses at different stages of their journey [31]. Both Mok [32] and Abuidhail [33] identify considerable gap exists between the level of expected support and the real support received by mothers and they felt that they were not supported by the healthcare team.

The present study showed that the NICU nurses were focused on doing complex clinical tasks delivery and relied heavily on maternal presence to monitor and perform primary care for their newborns under supervision of the nurses. The nurses appeared to believe that the mother was primarily responsible for the care; however this conflicted with their professional responsibilities; the nurses are ultimately responsible for the care and while supporting the mother with learning new parenting skills. The reliance of the nurses on maternal presence for primary care of newborns and being focused on technical tasks and interventions are consistent with other studies exploring Iranian NICU nurses' practice [34, 35]. However, this suggests is that the scope of nursing assessment, care planning, and delivery is arguably narrow. Restricting nursing practice to tasks and interventions limits the ability of the whole-person and family-centered care to be delivered.

In this context, Aein et al. [36] suggest that nursing focus on technical tasks is attributed to a shortage of nurses. Due to staffing ratios and high patient loads, nurses do not have enough time to support and engage in practical, meaningful communication with parents.

The findings from this study showed that mothers did not receive adequate information regarding their newborn’s clinical condition. Both medical and nursing staff did not appear to priorities spending time on communication purposes and sharing of information. It was considered by both medical and nursing staff a medical responsibility to discuss clinical details. Previous studies show that mothers of newborns admitted to the NICU need information about the health and condition of their newborn [37].

Although the need for information is a fundamental needs of the mothers who do not have sufficient knowledge and information about their newborn's disease [38]. One of the most important needs of mothers is clear communication and receiving honest answers to their questions [39]. A systematic review by Obeidat [9] showed that mothers of newborns admitted to the NICU face challenges such as access to information, disclosure of diagnosis, treatment, prognosis, and lack of control over their newborns care.

The process of obtaining information occurs through effective and meaningful communication. The information is communicated in a way that is specific, honest, timely, consistent, and intelligible. Providing mothers with adequate information is a well-recognised form of support [40]. Therefore, health professionals must engage in open communication with mothers across the continuum of care.

The majority of nurses in this study reported that the significant barrier to meeting mothers' support needs was a lack of staff. When staffing is optimized, nurses have the time to partner with mothers in care planning and delivery, identify and respond to their unique needs and provide the much-needed education/clinical explanations regarding the care of the newborn [41]. Increasing nursing demands combined with a workforce supply shortage are significant contemporary challenges for health systems globally [42]. Turner suggests that the nurses’ supportive role is dependent on elimination of the staffing shortages [43].

The findings of this study showed that nurses were not indifferent to the emotional stresses of mothers. There were times when emotional support was provided, such as when mothers could not cope due to reduced physical function or poor post-natal mental health. Mothers with low mood, anxiety, acute stress, and grief received a more significant emotional support from the nurses.

The researcher's observations in this unit showed that the nurses were not indifferent to the emotional stresses of mothers, and in situations where mothers experienced frustration, sadness, and grief caused by the newborn's illness, they supported them emotionally.

Studies conducted in Iran show that mothers who especially have abnormal newborns, experience high anxiety and stress, thus needing the emotional support of nurses [44, 45]. In Wigert's study, mothers in the neonatal intensive care unit received more emotional support from nurses [46]. In another study, emotional support was the most supportive of both parents [47]. Numerous studies on the mothers of premature newborns have shown that reassurance is essential to them [48]. Reassuring mothers helps them feel that they are being too talked as friends and that their role as a mother is respected [49]. In another study, nurses' reassurance was reported as one of the positive experiences of mothers of newborns admitted to the neonatal intensive care unit [50].

The present study showed that the nurses would not compel the mothers to take care of their newborns if they were not physically or emotionally ready to participate in the care delivery. Care support has also been confirmed in other studies in Iran. In the study of Mehdizadeh (2017), Seeydamini (2011), and Valizadeh (2009), the most support provided to mothers was in the care dimension [15, 51, 52]. It seems that the care of newborn is the nurses’ main responsibility and mothers’ participation in care has to be supported by the nurses.

In the current study, most of the information provided by the NICU health staff to mothers centered on newborn care. The content was explicitly focused on aspects of care mothers needed to provide as opposed to clinical information. Mothers expressed a desire for more excellent clinical information. This study showed that nurses played an influential role in mothers, which provides an opportunity for mothers to participate in this care. Mothers' education is a vital component of family-centered care [53]. According to previous studies, mothers' education has a supportive role for nurses [54], and without education and exchange of information, participation is impossible [55]. Educating mothers about newborn care techniques reduces mothers' fears about care and increases their participation [56]. In the Brach study, parental education also increased parents' ability to solve problems and their mental readiness to care for their newborns at home [53].

A study showed that the main nursing support among the mothers of newborns with congenital anomalies were related to their information needs about their newborn [45]. The previous studies showed that providing information to mothers in the intensive care unit leads to increased trust and participation of mothers [57, 58].

In this study, shortage of staffing and workload was identified as the main barriers to maternal support, and the employment of sufficient health care staff in the NICU is necessary. Also, family members and friends could be considered to support the mother in the NICU.

According to the results of this study, family-centered care, especially sharing information and support, is not yet fully implemented. It is recommended to conduct a study on barriers to the implementation of family-centered care in the NICU.

### Limitation

In this study, mothers were the primary care providers for their newborns, and due to cultural and religious beliefs and restricted physical space, fathers did not have a continuous presence in the NICU. Thus, we could not investigate fathers' support experiences in our study. However, the role fathers fulfilled in their newborn lives must be acknowledged.

## Conclusion

This study showed that, a gap exists between the level of expected support and the real support received by mothers. The health care staff activities were mostly patient-centered, focusing mainly on the treatment and care of infants, and the other principles of family-centered care such as support and information sharing have received less attention. Mothers' accompany in the care of their newborn should be under the nurses supervision and the mothers be informed about diagnosis, treatment, and prognosis of their newborn disease.

Support for mothers is not a priority for the health care team and is often provided in cases where mothers need support immediately. However, health care workers recognised maternal support as a necessity and described the workload as a barrier to maternal support at the NICU. Health care staff support for mothers was not a priority and was provided especially when mothers need immediate support. However, the health care staff confirmed meeting the supportive needs of mothers as a necessity, they considered workload as a barrier to maternal support in NICU.

## Data Availability

The datasets generated and/or analysed during the current study are in Persian language and are not publicly available due to confidentiality of the participants, but are available from the corresponding author on reasonable request.

## Abbreviations

NICU: Neonatal intensive care unit
FCC: Family-centered care
